# Hypertension Awareness and Associated Factors among Older Chinese Adults

**DOI:** 10.3389/fpubh.2013.00067

**Published:** 2013-12-06

**Authors:** SangNam Ahn, Matthew Lee Smith, Jinmyoung Cho, James E. Bailey, Marcia G. Ory

**Affiliations:** ^1^Division of Health Systems Management and Policy, School of Public Health, The University of Memphis, Memphis, TN, USA; ^2^Department of Health Promotion and Community Health Sciences, Texas A&M Health Science Center School of Rural Public Health, College Station, TX, USA; ^3^Department of Health Promotion and Behavior, College of Public Health, The University of Georgia, Athens, GA, USA; ^4^Center for Applied Health Research, Scott & White Healthcare, College Station, TX, USA; ^5^Department of Medicine, University of Tennessee Health Science Center, Memphis, TN, USA

**Keywords:** hypertension, awareness, elderly care, rural, Asia, Chinese

## Abstract

Hypertension is one of the most preventable chronic conditions. Improving hypertension awareness is a critical first step to reducing morbidity and mortality from hypertension in the elderly, yet the factors associated with hypertension awareness in China are poorly understood. The objective of this paper is to examine the extent to which older Chinese adults are aware of their hypertension, and factors associated with this awareness. We included 2404 adults aged 60 years or older clinically identified as hypertensive from panel data surveyed in 1997, 2000, 2004, and 2006 as part of the China Health and Nutrition Survey. Comparing this data with respondents’ self-reported diagnosis of hypertension enabled us to characterize hypertension awareness. Covariates included socio-demographic, health status, functional disability, and behavioral factors. Generalized estimating equations were used to identify factors for hypertension awareness. We found 22.9% in 1997 and 42.7% in 2006 of study participants were aware of their hypertensive status. Lower awareness was found among those who lived in rural areas [odds ratio (OR) = 0.64, 95% Confidence Interval (CI), 0.47–0.88]. Higher awareness was noted for persons who were aware of their hypertensive status in a previous survey wave (OR = 7.43, 95% CI, 5.45–10.13), had high income (OR = 1.55, 95% CI, 1.05–2.28), had stage two hypertension (OR = 2.28, 95% CI, 1.69–3.06), had acute condition (OR = 2.54, 95% CI, 1.89–3.42), and had greater activities of daily living limitations (OR = 1.24, 95% CI, 1.08–1.43). Studying dynamics of hypertension awareness can help inform both clinical and public health approaches to improve healthcare.

## Introduction

As its economy has dramatically grown over the past 30 years, China has undergone profound changes in disease prevention and control including increased vaccination coverage, better sanitation, and improved access to medical care (1). However, the rapidly aging population coupled with accelerated urbanization places older adults in greater jeopardy of health disparities and inadequate access to health care services (2, 3). Improvements in household income have also given rise to Western ailments such as obesity, diabetes, and hypertension, which are associated with Western lifestyles characterized by high calorie and dietary fat intake and low levels of physical activity (4).

Hypertension is one of the most preventable chronic conditions (5), but each year is responsible for 7.6 million premature deaths and the loss of 92 million disability-adjusted life years worldwide (6). Hypertension presents challenges not only to individual health but also to healthcare delivery and financial systems in China (1). The burdens of hypertension are more pronouncing older adults (7). Furthermore, the prevalence of diagnosed hypertension among Chinese adults aged 65 years and older has increased rapidly to 64% in urban and 57% in rural areas (8).

For the purposes of this study, hypertension awareness is defined as a self-report of being diagnosed by a care provider as having hypertension (9). Improving hypertension awareness is a critical first step to reducing morbidity and mortality from hypertension in the elderly, yet the factors associated with hypertension awareness in China are poorly understood. Unawareness or under-awareness of hypertension may be attributed to the lack of routine blood pressure (BP) screenings and the absence of hypertension-related education programs among this population (especially in rural areas) (10). Lack of awareness about one’s own hypertension has been associated with failure to receive high quality care (11), and leads to lost opportunity to prevent more severe cardiovascular-related conditions (12). Although most hypertensive patients in the U.S. are aware of their hypertension (13), in developing countries like China rates of hypertension awareness are still far from optimal. More than 80% of U.S. older adults were aware of their hypertension in 2008 (13); however, only 44% of older Chinese adults were aware of their hypertensive status in 2004 (10).

In the U.S., individuals with lower hypertension awareness have been identified as being male, perceiving their health as good to excellent, and lacking awareness about their parents’ history of high BP (14). Although previous surveys have demonstrated factors associated with hypertension awareness in the Chinese population (e.g., married status, female gender, and prior hypertension screening), past studies have not focused on the high-risk elderly population (10). A recent study suggests that lack of hypertension awareness in older Chinese adults is related to limited access to regular medical check-ups and poor knowledge about hypertension (15). A prior study in a multiethnic Asian population found that reduced awareness was associated with being younger, never married, and having higher education level (16). Nevertheless, lack of hypertension awareness among Chinese older adults has garnered little attention even though China is aging rapidly and will experience substantial demand for healthcare services in forthcoming years (1). This study aims to (a) examine the extent to which older Chinese adults are aware of their hypertensive status, and (b) explore factors associated with hypertension awareness.

## Materials and Methods

### Data source and study population

Longitudinal data for this study was obtained from the China Health and Nutrition Survey (CHNS). The CNHS was conducted in 1989, 1991, 1997, 2000, 2004, and 2006 and designed to study how social and economic transformation influenced the health and nutritional status of residents in nine provinces of China that varied substantially in geography, economic development, public resources, and health status (17). The survey used a multistage, random cluster process to draw a sample from each province to capture a range of demographic and economic circumstances (18). Counties and cities in each province were stratified by income (low, middle, and high), and a weighted sampling scheme was used to randomly select four counties and two cities in each province (18). Villages and townships within the counties and urban and suburban neighborhoods within the cities were selected randomly (18). Through this process, a sample of 216 communities from nine provinces was obtained, which comprised 36 urban neighborhoods, 36 suburban neighborhoods, 36 towns, and 108 villages (18). The household sample was 4020 in 1989 and 4467 in 2006. For individuals, the sample was 15,927 in 1989 and 18,764 in 2006 (18). We used all available data from adults aged 60 years or older who were surveyed at least once in the 1997, 2000, 2004, and 2006 survey waves using methods similar to those employed in our previous studies of Chinese older adults using the CHNS (19). Only hypertensive patients were included in the current study. BP was measured three times on one visit during each survey, which were averaged and reported as the BP values in this study (20). Being hypertensive was defined as those with the mean systolic BP of at least 140 mm Hg, the mean diastolic BP of at least 90 mm Hg, and/or self-reported use of prescription medication to lower BP (13). Hypertension was further classified into stage 1 (systolic 140 mm Hg or above or diastolic 90 mm Hg or above) and 2 (systolic 160 mm Hg or above or diastolic 100 mm Hg or above) (21). Our final analytic sample size was 2404 participants.

### Measures

#### Dependent variable

Hypertension awareness was assessed through self-report using the following question, “Has a doctor ever told you that you suffer from high blood pressure? (13)” Hypertensive participants who responded affirmatively to this question were deemed to be aware of their hypertension.

The following independent variables were selected from the CHNS dataset that had been previously demonstrated in the literature to be associated with hypertension awareness.

#### Socio-demographic factors

Household income was computed as the sum of all sources of income in the household, inflated to a 2006 price index, and divided by household size. The current study examined tertiles of per capita income based on the distribution of the continuous form of this variable in the selected study sample (17). Rural-urban area of residence is based on a classification of the 190 primary sampling units as being “urban” or “rural communities” and the detailed measurement procedures are described in a previous publication (17). The education level was trichotomized based on completed years of education: none, primary, and intermediate or more education (17). Marital status was classified as never married, married, divorced, widowed, and separated, collapsing into two categories: living with a spouse or living without a spouse. Participants also reported their sex (male or female) and age (continuous range from 60 to 101 years).

#### Health status

Body mass index (BMI) was calculated by dividing weight in kilograms by the square of height in meters and rounding to the nearest tenth as recommended by the original Quetelet calculation (22). Participants were asked to report if they had experienced any of the following acute symptoms during the last 4 weeks: (a) fever, sore throat, cough; (b) diarrhea, stomachache; (c) headache, dizziness; (d) joint pain, muscle pain; (e) rash, dermatitis; (f) eye/ear disease; (g) any other infectious disease; and (h) other non-communicable diseases (17). These responses were aggregated and then dichotomized into yes or no for distribution characteristics. Cognitive function was measured by a single self-reported question regarding memory status (17). Those who responded “bad or very bad” were considered to have a possible cognitive impairment relative to peers who answered “very good, good, okay (17).” Functional limitations were evaluated using number of activities of daily living (ADL) (ranging 0–6) and instrumental activities of daily living (IADL) limitations (ranging 0–5). ADL limitations were based on such items as bathing, dressing, eating, grooming, toileting, and walking (23) while IADL limitations included shopping, cooking, using public transportation, managing money, and using the telephone (24).

#### Lifestyle behaviors

Respondents were asked their current smoking and drinking alcohol status (yes or no). Physical activity was based on non-leisure physical activity levels (25): having no physical activity, or engaging in very light (e.g., office worker), light (e.g., driver), heavy (e.g., farmer), or very heavy physical activity (e.g., miner). We classified these activities into no or very light, light, and moderate or heavy physical activity.

#### Health insurance

Health insurance was coded as a binary variable (yes or no) through self-report using the following question, “Do you have medical insurance?”

### Statistical analysis

All analyses were conducted with the Stata (version 11) statistical package (26). We used generalized estimating equations (GEE) to estimate population-averaged (marginal) effects considering the dependence among individuals nested in survey clusters (27) and also to describe how the entire population evolved over time (28). With an exchangeable correlation structure, we applied a robust variance estimator, which is always consistent even when the covariance structure is misspecified (27). We also included the previous (lagged) response *y*_*i,t*−1_ as a covariate in the final model to partially control stochastic time-varying covariates (27). For model selection, we used quasilikelihood under the independence model criterion, which helps select the best working correlation structure in GEE analyses (29). Trends in hypertension awareness over time were assessed for statistical significance using multivariate tests on means assuming multivariate normality. Odds ratios (ORs) with 95% confidence intervals (CIs) are displayed.

## Results

Table 1 shows the number of study participants in each survey period (i.e., 936 in 1997, 830 in 2000, 1019 in 2004, and 1048 in 2006). The characteristics of the study participants from 1997 to 2006 are presented in Table 1. Study participants who were aware of their hypertension status ranged between 22.9% in 1997 and 42.7% in 2006 (*P* < 0.001). The proportion of participants reported having high income ranged between 17.0% in 1997 and 42.7% in 2006 (*P* < 0.001). The average age of the sample was 70 years. The sample contained larger proportions of participants had no formal education (*P* < 0.001) and were living with a spouse (*P* = 0.010). For health conditions, in 2006, 58.5 and 41.5% were classified into stage 1 and 2 hypertension, respectively (*P* < 0.001). Study participants who reported having one or more acute conditions ranged between 12.9% in 1996 and 41.0% in 2006 (*P* < 0.001). Study participants reported having on average 0.5 ADL limitations (0.46 in 1997 and 0.54 in 2006) (*P* = 0.002). Participants also reported having health insurance ranging between 31.1% in 1997 and 53.3% in 2006 (*P* < 0.001).

**Table 1:** **Characteristics of study participants in the China health and nutrition survey (*n* = 2404) from 1997 to 2006**.

Variable		% or Mean (SD)				*P*
		1997 (*n* = 936)	2000 (*n* = 830)	2004 (*n* = 1019)	2006 (*n* = 1048)	
Hypertension awareness		22.9	33.7	34.9	42.7	<0.001
Income	Low	46.3	37.6	25.7	29.8	<0.001
	Medium	36.7	33.0	30.6	27.5	
	High	17.0	29.4	43.7	42.7	
Age		69.5 (7.1)	69.6 (6.8)	70.2 (7.0)	70.3 (7.3)	<0.001
Sex	Female	54.8	52.4	53.7	52.9	0.748
Rural residence	Rural	59.3	58.2	60.0	63.3	0.120
Education	No school	56.1	49.9	37.5	40.3	<0.001
	Primary	29.7	32.9	38.2	32.2	
	≥Intermediate	14.2	17.3	24.2	27.5	
Marital status	Living with a spouse	64.5	67.8	68.3	71.5	0.010
	Living without a spouse	35.5	32.2	31.7	28.5	
Hypertension category^a^	Stage 1	50.1	60.8	56.9	58.5	<0.001
	Stage 2	50.0	39.2	43.2	41.5	
Body mass index (kg/m^2^)		23.3 (3.9)	23.9 (4.0)	24.0 (4.4)	24.1 (4.2)	0.013
Acute condition		12.9	22.3	42.2	41.0	<0.001
Memory deterioration	Same or improved	52.9	51.5	49.9	44.5	0.002
	Deteriorated	47.1	48.5	50.1	55.5	
ADL limitation^b^		0.46 (1.2)	0.46 (1.2)	0.46 (1.3)	0.54 (1.4)	0.002
IADL limitation^c^		1.3 (1.6)	1.1 (1.5)	1.1 (1.6)	1.1 (1.7)	0.132
Smoking	No	74.9	74.6	77.2	78.9	0.085
	Yes	25.1	25.4	22.8	21.1	
Alcohol	No	74.6	71.6	71.6	74.6	0.222
	Yes	25.5	28.4	28.4	25.4	
Physical activity	No or very light	54.8	55.0	55.5	56.8	0.776
	Light	22.1	20.7	22.0	19.5	
	≥Moderate	23.1	24.4	22.5	23.7	
Health insurance		31.1	26.6	30.8	53.3	<0.001

*^a^ Defined by American Heart Association*.

*^b^ Activities of daily living*.

*^c^ Instrumental activities of daily living*.

Table 2 displays factors associated with awareness of hypertension. Hypertension awareness was observed more among those who were aware of hypertension in the previous survey wave (OR = 7.43, *P* < 0.001), reported having a high income (OR = 1.55, *P* = 0.026), being in 2006 cohort (OR = 1.43, *P* = 0.040), having stage 2 hypertension (OR = 2.28, *P* < 0.001), and having greater ADL limitations (OR = 1.24, *P* = 0.003). Conversely, hypertension awareness was observed less among those who lived in rural areas (OR = 0.64, *P* = 0.006).

**Table 2:** **Factors associated with hypertension awareness in older Chinese adults (*n* = 849)^a^**.

Variable		OR^b^	*P*	95% CI	
Lagged awareness (*y_t_*_−1_)		7.43	<**0.001**	5.446	10.125
Income	Low	1.00	–		
	Medium	0.99	0.963	0.677	1.450
	High	1.55	**0.026**	1.054	2.283
Age		0.99	0.373	0.964	1.014
Sex	Male	1.00	–		
	Female	1.25	0.221	0.873	1.802
Rural residence	Urban	1.00	–		
	Rural	0.64	**0.006**	0.466	0.877
Education	No school	1.00	–		
	Primary	1.34	0.106	0.939	1.918
	≥Intermediate	1.30	0.266	0.820	2.052
Marital status	Living with a spouse	1.00	–		
	Living without a spouse	0.93	0.629	0.674	1.269
Cohort	1997	1.00	–		
	2000	1.21	0.353	0.812	1.792
	2006	1.43	**0.040**	1.017	2.022
Hypertension category^c^	Stage 1	1.00	–		
	Stage 2	2.28	<**0.001**	1.692	3.064
Body mass index (kg/m^2^)	1.03		0.084	0.996	1.069
Acute condition	No	1.00	–		
	Yes	2.54	<**0.001**	1.885	3.424
Memory deterioration	Same or improved	1.00	–		
	Deteriorated	1.16	0.349	0.853	1.569
ADL limitation^d^		1.24	**0.003**	1.078	1.432
IADL limitaiton^e^		0.98	0.700	0.866	1.101
Smoking	No	1.00	–		
	Yes	1.07	0.713	0.745	1.537
Alcohol	No	1.00	–		
	Yes	0.85	0.389	0.587	1.231
Physical activity	No or very light	1.00	–		
	Light	0.94	0.771	0.633	1.404
	≥Moderate	0.79	0.327	0.498	1.261
Health insurance	No	1.00	–		
	Yes	1.05	0.780	0.761	1.438

*^a^ Generalized estimation equation model*.

*^b^ Odds ratio*.

*^c^ Defined by American Heart Association*.

*^d^ Activities of daily living*.

*^e^ Instrumental activities of daily living*.

*Bold numbers are statistically significant*.

## Discussion

Overall, the proportions of respondents who were aware of their hypertensive status in this study based on CHNS ranged between 22.9% in 1997 and 42.7% in 2006. In other words, 77.1% in 1997 and 57.3% in 2006 were unaware of their hypertension. This level of awareness of hypertension among older Chinese adults is lower than an awareness level suggested by Muntner and colleagues analyzing the InterAsia data in a prior study: 44.1% (≥65 years) (10). This difference probably comes from different age profile and study periods: CHNS vs. InterAsia (≥60 vs. ≥65 years; 1997–2006 vs. 2000–2001). Older study participants (≥65 years) at the InterAsia study than CHNS may have higher prevalence of hypertension and may tend to be aware more of their hypertensive condition compared to younger counterparts in the current study.

The current study showed significant and favorable increases in hypertension awareness among older Chinese adults from 22.9% in 1997 to 42.7% in 2006. Given that the new health system reform started 2009 (30), it is impossible to connect older Chinese adults’ hypertension awareness improvements in the current study with the new universal health care system. In spite of the improving hypertension awareness, the levels of hypertension awareness (i.e., 42.7%) seen in the 2006 CHNS survey are still very concerning and mirror hypertension awareness levels not seen in the U.S since the 1960s (13). A recent NHANES survey in the U.S. demonstrated hypertension awareness above 80% among those aged 60 years or older (13). The low levels of hypertension awareness seen in the Chinese elderly population are particularly problematic given the increasing prevalence of hypertension in this group. Lack of hypertension awareness has been identified as one of the public health and medical challenges in the prevention and treatment of the modifiable disease (31). The current study identifies important factors associated with hypertension awareness among older Chinese adults. We found that being aware of hypertension was associated with higher income level, residing in urban areas, and various health conditions.

Lack of hypertension awareness is a major problem in low-income countries (32). Consistent with previous results identifying the high cost of antihypertensive medication as a primary barrier for better addressing hypertension (32), the current study suggests a positive association between income and hypertension awareness. Study participants with higher incomes were 55% more likely to be aware of their hypertension compared to those with lower incomes. As a supplemental analysis (not shown at the Tables), we also examined how mean income and hypertension awareness varied over the survey periods. Increases in hypertension awareness paralleled a dramatic increase in per capita household income of study participants from 3333 Yuan in 1997 to 4482 Yuan in 2000, 6500 Yuan in 2004, and 6808 Yuan in 2006 ($1 USD ≈ 6.2 Yuan as of January 2013). Over the same period of time, study participants’ hypertension awareness significantly increased from 22.9% in 1997 to 33.7% in 2000, 34.9% in 2004, and 42.7% in 2006. These findings suggest that higher income levels may enable older adults to have better access to health care services and routine check-ups, which is consistent with previous studies (33). Nevertheless, further study is required to examine how income is associated with hypertension awareness among older Chinese adults.

Rural residence was associated with 36% lower hypertension awareness. We posit that lower income and fewer healthcare resources in Chinese rural areas mediate the relationship between rurality and hypertension awareness. This is due, in part, to the existence of disparities in health services utilization in China. For example, only 7.6% of rural residents use hospital services compared with 11.1% of urban residents in China (34). The Chinese rural-urban divide is fairly pronounced in light of healthcare and financial resources. More than 65% of older Chinese adults in rural areas who did not seek healthcare cited excessive costs and inability to pay for prescription drugs as primary reasons for not accessing the healthcare system (35). It is also noteworthy that the accelerated migration of younger adults, who used to informally care for their older parents, has resulted in more older Chinese adults being left behind to live in rural areas and having higher levels of depression (36). Given these social changes, it is relevant to cite two recent studies regarding suicidal ideation/attempts and utilization of health care services among older adults living in rural areas. Lifetime suicidal thoughts and attempts among older Chinese adults in rural areas were 28.9 and 5.3%, respectively, compared to their urban (Beijing) counterparts (4.8, 1.9%, respectively) (37). Another study also found that people covered by the New Rural Cooperative Medical Scheme, a health care reform launched in 2003, were more likely to underutilize outpatient care than the uninsured because of low household incomes (38). Given that ever rising regional economic inequality is known to contribute to regional health disparities (39), the establishment of medical relief policies, and chronic disease management programs for these vulnerable populations (including older adults in rural areas) might be especially warranted.

Contrary to our expectations, health insurance did not affect the presence of hypertension awareness. We found an increasing trend of having health insurance between 31.1% in 1997 and 53.3% in 2006, which is still suboptimal level of access to healthcare. These statistics may resonate with the current Chinese healthcare establishment where previous studies found 43% of urban populations lacked health insurance in 2003 and 87% of rural populations lacked health insurance in 1999 (35). Due to the recent universal health care efforts, basic clinical health care has covered 90% of rural population; however it is reported that the low level of premiums of the rural health insurance scheme constrained scope of services and financial protection (30). The results of the current study raise the question of why having health insurance was not associated with hypertension awareness among older Chinese adults. Given that an increase in the ability to pay is significantly associated with a higher likelihood of utilizing healthcare (40), we postulate that study participants may not be able to access healthcare services due to high out-of-pocket costs. In 1990, Chinese households paid for 36% of healthcare expenditures out-of-pocket, but this figure increased up to 58% in 2002 (41). This could be part of the reason many Chinese people blame the government for a worsening healthcare system that has eschewed its’ responsibility for running hospitals and shifted urban care to a cost-sharing insurance scheme (35). This issue substantially contributed to excluding rural-urban migrant workers from full participation in the healthcare system (35). Further study is warranted to examine the effects of how China’s new health care reform has affected the awareness of chronic conditions (especially hypertension).

The current study also demonstrated a positive relationship between poor health status and hypertension awareness. Participants tended to be accurately aware of their hypertensive status when they had severe hypertension (stage 2), had acute conditions, and had greater functional limitations. The most plausible explanations for these associations will be that patients with poor health conditions will tend to seek for medical assistance and, in turn, they became aware of their hypertensive status. A prior study found that the odds of being aware of hypertension increased among those who were diagnosed of having diabetes and had a regular physician visit, while the odds decreased among those who perceived their health as good to excellent (14). These findings raise interesting public health questions: what if people who have hypertension but are not aware of it cannot get access to healthcare providers because of their rural residence and lack of health insurance. Certainly warranted are further studies on which factors can mediate the relationship between health status and hypertension awareness under the new health care reform in China.

As in any research, this study has some limitations to consider. First, the CHNS sample was not designed to be representative of China, and we are cautious about generalizing these study findings. Second, the sample size of analyzed cases was substantially reduced because of methods used to control for time-varying covariates in the model. Introducing the previous (i.e., lagged) response variable and variables from the 2004 wave dataset resulted in multicollinearity. Thus, the sample size reduced from 2404 in Table 1 to 849 in Table 2. Third, the study findings should be cautiously interpreted because of relatively outdated dataset and in the context of the new health care reform in China. Fourth, the current study included a single item related to health insurance, which may have limited our ability to capture the complexities of the Chinese health insurance system. Fifth, we may not be able to control the temporal changes of independent variables over the survey periods with 9 years between the first and last survey waves. Despite these limitations, the current study has value in examining factors related to hypertension awareness among older adults in a rapidly industrializing and aging society. Given the prevalent unawareness of hypertension among the Chinese elderly population, we recommend more direct measurements and diverse methods to increase hypertension awareness among high-risk older adults in this fast-developing country. This is important because adequate treatment and control of hypertension relies heavily on accurate diagnosis (42). Furthermore, it is worthwhile to document hypertension awareness among older adults after the 2009 China health systems reform.

In conclusion, the current study found positive associations between hypertension awareness and higher income, urban residence, and having various health conditions. Clinicians should take special note of older at-risk patients to ensure screening and monitoring of hypertension on a regular basis. Policy makers should identify which subgroup populations are consistently unaware of their hypertensive status and provide tailored interventions and treatment to achieve better control of hypertension. This study suggests that lower income rural residents in China should be particularly targeted by hypertension screening and awareness campaigns. Hypertension awareness can be also improved by increasing public knowledge and health education targeting general public.

## Authors Contribution

SangNam Ahn planned the study, analyzed the data, and wrote the article. Matthew Lee Smith assisted in data interpretation and in critical revision. Jinmyoung Cho assisted in data interpretation and wrote the article. James E. Bailey assisted in data interpretation and provided critical revisions. Marcia G. Ory conceived and supervised the study, and provided critical content and revisions.

## Conflict of Interest Statement

The authors declare that the research was conducted in the absence of any commercial or financial relationships that could be construed as a potential conflict of interest.
